# Tips and nodes are complementary not competing approaches to the calibration of molecular clocks

**DOI:** 10.1098/rsbl.2015.0975

**Published:** 2016-04

**Authors:** Joseph E. O'Reilly, Philip C. J. Donoghue

**Affiliations:** School of Earth Sciences, University of Bristol, Life Sciences Building, Bristol BS8 1TQ, UK

**Keywords:** molecular clock, calibration, tip, node, Hymenoptera

## Abstract

Molecular clock methodology provides the best means of establishing evolutionary timescales, the accuracy and precision of which remain reliant on calibration, traditionally based on fossil constraints on clade (node) ages. Tip calibration has been developed to obviate undesirable aspects of node calibration, including the need for maximum age constraints that are invariably very difficult to justify. Instead, tip calibration incorporates fossil species as dated tips alongside living relatives, potentially improving the accuracy and precision of divergence time estimates. We demonstrate that tip calibration yields node calibrations that violate fossil evidence, contributing to unjustifiably young and ancient age estimates, less precise and (presumably) accurate than conventional node calibration. However, we go on to show that node and tip calibrations are complementary, producing meaningful age estimates, with node minima enforcing realistic ages and fossil tips interacting with node calibrations to objectively define maximum age constraints on clade ages. Together, tip and node calibrations may yield evolutionary timescales that are better justified, more precise and accurate than either calibration strategy can achieve alone.

## 1. Introduction

The molecular clock has displaced the fossil record as the primary means of establishing an evolutionary timescale; however, the accuracy and precision of divergence time estimates and their fossil calibrations remain inextricably linked [1]. Traditionally, divergence time estimation has achieved calibration based on geological (usually palaeontological) constraints on clade (node) ages. This approach has been developed to the extent that further improvements in accuracy and precision are limited by the inherent uncertainty in fossil evidence. Indeed, it is this uncertainty that has called into question the approach of node calibration, particularly what some see as the over-interpretation of palaeontological data to establish maximum constraints on clade ages, and the difficulty in objectively representing prior evidence of node age as a probability distribution [2]. Furthermore, node age constraints invariably differ from those specified as a consequence of their integration into the joint time prior on node ages [3]. These concerns have led to the replacement of node calibrations with tip calibrations in which fossil species of a known age are integrated directly into divergence time analyses, supplementing sequence data from living species with morphological data from living and fossil species [2,4]. However, there has been little effort to demonstrate the effect of different approaches to calibration and, indeed, to determine whether the effective prior on node ages resulting from tip calibration is compatible with the fossil evidence usually employed in node calibration. This is of particular interest given growing concern that tip calibration consistently yields unrealistically ancient divergence time estimates [5].

Hence, we sought to compare the efficacy of tip and node calibrations by determining the compatibility of the resulting effective prior on node ages resulting from tip calibration and fossil-based node age constraints. This is readily sampled in node- and tip-calibrated analyses when the time prior is conditioned on a fully constrained topology upon which ages are estimated. However, it is challenging where topology and time are coestimated. Here, we show that, in such circumstances, an approximation of the time prior can be obtained by conditioning on the consensus tree derived from a posterior sample of trees. Using an empirical dataset, we show that effective node age priors derived from tip calibration are often incompatible with fossil evidence, violating either minimum or maximum node age constraints. We argue that this contributes to the unrealistically ancient divergence time estimates produced by tip calibration. These artefacts are diminished by combining tip and node calibrations, where node calibrations ensure that divergence time estimates never violate fossil-based minima and tip calibrations effectively establish node age maxima.

## 2. Material and methods

We compared the effective node age priors and posteriors for tip and node calibrations using a previously published hymenopteran dataset of molecular and morphological characters [2]. The original study assumed errorless tip-ages for fossil species. We employed revised ages for these species, integrating associated uncertainty and derived node age constraints in order to compare effective priors on node ages to the palaeontological evidence [5]. Uncertainty in fossil taxon age was represented with uniform distributions, whereas node calibrations were assigned offset exponential distributions, as in [2]. Unbounded distributions allow maxima to be defined by interaction between node and tip calibrations.

To obtain an approximation of the time prior, we sampled from the prior while conditioning on the consensus of a sample from the posterior distribution of trees obtained from a standard tip-calibrated total evidence dating (TED) analysis. We then constrained the topology to the consensus tree and sampled from the prior conditioned on this tree, providing a meaningful approximation of the effective time prior in a topologically unconstrained tip-calibrated analysis (electronic supplementary material methods).

To evaluate the influence of tip calibrations, we compared effective priors and posterior estimates of node ages from tip-calibrated analysis to the raw palaeontological constraints on node ages, and to the effective priors and posterior estimates of node ages derived from (i) a node-calibrated analysis and (ii) an analysis that implemented both tip and node calibrations. In the latter, fossil taxa were assigned to clades identified in the standard tip-calibrated analysis; where possible, the clades were assigned node calibrations. Minima on node-calibrated clades are defined by fossil evidence and maxima are established based on interaction between node and tip calibrations. We obtained a posterior sample of trees using the consensus tree produced from this sample to sample from the effective time prior. Several fossil taxa and node calibrations could not be included in this analysis because of limitations of MrBayes (see the electronic supplementary material for detail).

## 3. Results

**Figure 1.**
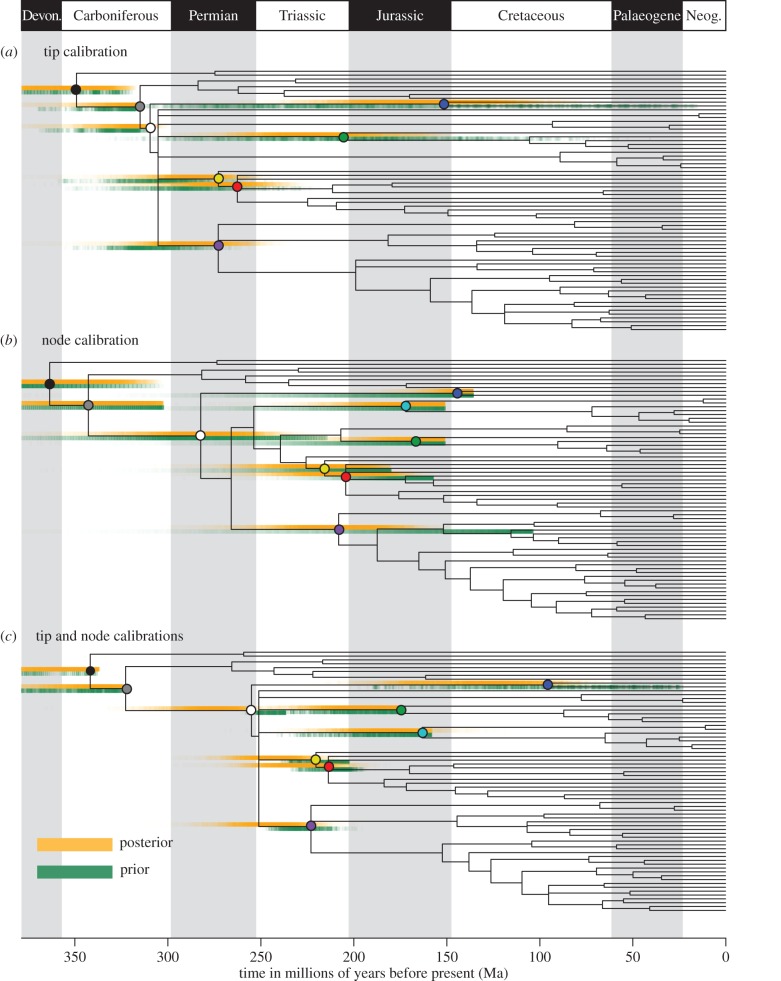
Time-calibrated phylogenies of Hymenoptera based on: (*a*) tip calibration, (*b*) node calibration and (*c*) combined tip and node calibrations. Panels (*a*,*c*) are presented with fossil taxa removed, complete topologies are presented in the electronic supplementary material. Graduated bars represent the prior and posterior distribution of clade age, with colour density correlated with probability. Polytomies reflect topological uncertainty in the tree sample and are not indicative of simultaneous divergence. Coloured nodes indicate the position of the nine clades of interest across the three topologies. Black (Neoptera), grey (Holometabola), white (Hymenoptera), yellow (Vespina), red (Apocrita), purple (Tenthredinoidea), blue (Xyelidae), turquoise (Pamphilioidea) and green (Siricoidea).

Our tip-calibrated consensus topology (figure 1*a*) differs from [2] in the placement of fossil Xyelidae, which could not be resolved in our analysis. *Spathoxyela* and *Mesoxyela* form a polytomy with extant Xyelidae, because they are alternately assigned to crown or total-group Xyelidae in the tree sample; in the original analysis, all fossil Xyelidae were resolved to the stem in the consensus tree. Following [2], *Eoxyela*, the fossil defining the node calibration for Xyelidae, is resolved outside of crown Xyelidae. A number of fossil taxa, including *Palaeathalia* and *Cleistogaster*, were placed with higher resolution in our recalibrated analysis than in the original*.* Similar to [2], we were unable to recover unequivocal monophyly of Pamphilioidea.

**Figure 2.**
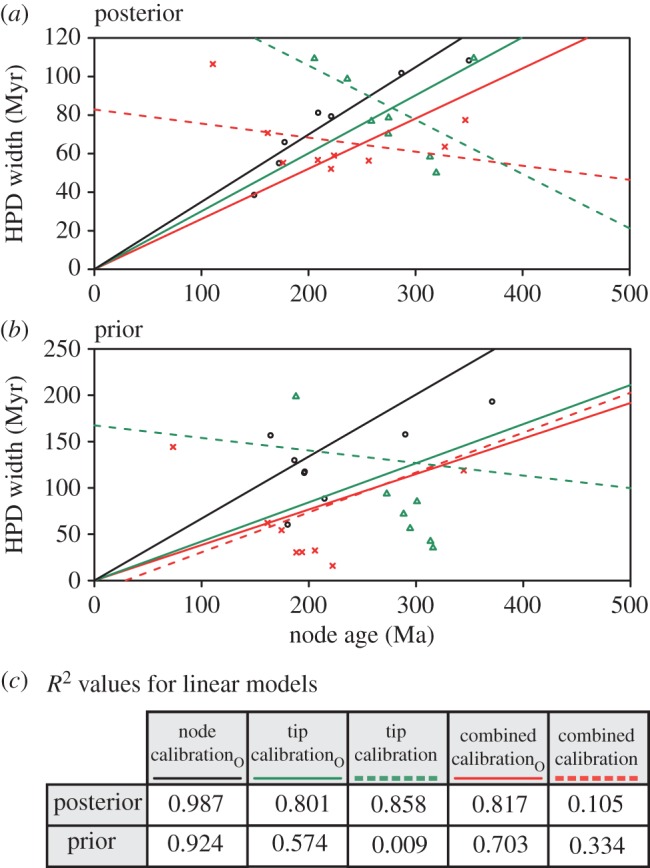
Infinite-sites plots [1] for three alternative calibration approaches for both the posterior (*a*) and prior (*b*) distribution of times of nine clades for which node calibrations could be applied. Solid lines represent the fitted linear model for each independent set of node ages when forced through the origin, as in [1]. Dotted lines represent the linear model for non-node-calibrated analyses when not forced through the origin, demonstrating the lack of a linear decrease in clade age confidence interval width. The fit of linear models is presented in (*c*). Models forced through the origin are indicated with a subscript O.

The effective priors on node ages resulting from tip calibration alone (excepting the two deepest nodes) consistently extend beyond the maximum palaeontological constraints on node ages, and include more ancient ages than the effective priors on node ages in the node-calibrated analysis. In two clades (Xyelidae and Siricoidea), tip calibration produces effective priors extending to the near Recent. The effective time priors on these clades plus Pamphilioidea extend beyond the minimum palaeontological constraints on the ages of these crown clades, and encompass younger ages than the effective priors on node ages in the node-calibrated analysis (figure 1*b*). In all instances, these differences propagate to the posterior estimates of clade ages. The anticipated linear relationship between node age and highest posterior density (HPD) width holds only for the node-calibrated analysis (figure 2). The results of the tip-calibrated analysis exhibit an inverse relationship, with uncertainty decreasing with proximity to the root.

When tip and node calibrations are combined (figure 1*c*), the effective priors on node ages encompass dates younger than the minimum palaeontological constraints on the ages of crown Pamphilioidea and crown Xyelidae; in all other clades, the effective priors and posterior age estimates fall fully within their palaeontological node age constraints. In all but the two deepest nodes the means of posterior estimates of clade age are consistently and significantly younger than their counterparts when only tip calibrations are implemented. The distributions of posterior estimates of clade age are also more precise than their tip-calibrated counterparts in all but the two most basal clades.

## 4. Discussion

It has been accepted generally that, because user-specified node age priors are truncated in construction of the joint time prior, the effective prior should be assessed to determine whether it is consistent with the palaeontological constraints [3]. Our results indicate that this approach should be extended to tip calibration. Tip calibrations consistently yielded older effective priors on node ages and older divergence time estimates. This occurs principally because of an absence of constraints on the ages of internal nodes within the tree, normally provided by node calibrations, allowing uncertainty to propagate from the tips, constrained only by the prior on the root age, skewing the distribution of prior probability towards ancient ages. We cannot conclude that these estimates are inaccurate merely because they are incompatible with palaeontological maximum age constraints. However, the effective priors derived from tip calibration of some node ages are younger than their palaeontological minimum age constraints, which is unreasonable. This occurs because some crown clades (Xyelidae, Pamphilioidea) in the tree sample are often resolved without fossil members and so their minimum ages are bounded only by the Recent.

The node-calibrated analysis is compatible with the palaeontological constraints on clade ages, because they are implemented as node calibrations. However, the combined node and tip-calibrated analyses yielded younger effective priors and posteriors than exclusively tip- or node-calibrated analyses, while also conforming to the palaeontological minimum constraints. This is clear in the case of Siricoidea, where no fossil member of the crown clade is represented but the zero-time constraint on the age of this clade in the tip-calibrated analysis is supplemented by a node age constraint in the combined tip- and node-calibrated analyses. The divergence time estimates derived from combined calibration are consistently younger—a consequence of the tip calibrations which act to truncate the broad priors of the node calibrations, extending from their hard minimum age constraints. This serves to draw the effective prior probability closer to the minima in the joint time prior, which propagates to the posterior divergence time estimates. In effect, the tip and node calibrations interact to operationally establish maxima for the node calibrations.

It is reasonable to question whether tip and node calibrations should be implemented together and, certainly, the same data should not be represented in both calibration methods. However, there is no logical inconsistency between these approaches, and some fossil data are better represented as a tip calibration or as a node calibration. While it has been argued that tip calibration facilitates the inclusion of all fossil species in divergence time analyses [2,4], some fossil taxa are too incomplete to be effective tip calibrations, but may be no less definitive in circumscribing the minimum age of a clade (e.g. the minimum ages of angiosperms and echinoderms are constrained by tricolpate pollen and fragments of stereom, respectively).

A casualty of the implementation of node calibrations in MrBayes is the ability to perform coestimation of time and topology, a particular advantage of the tip calibration approach [2]. However, fossil taxa are not commonly well-resolved through coestimation, a consequence of the paucity of morphological data and the non-random distribution of missing data for fossil species [5]. These challenges may be overcome simply by introducing a backbone of partial topological constraints, facilitating coestimation, but within the qualified phylogenetic uncertainty that is associated with most fossil species. Only beast is currently capable of fully accommodating this approach to combined calibration [6]. In our combined tip- and node-calibrated analyses, we were forced to exclude any fossil species whose age overlapped or extended beyond the node calibration for the clade to which it was assigned. This limitation occurs because MrBayes unnecessarily considers ages for fossil species that can be older than their assigned clade, yielding a negative clock-rate and, therefore, an error when calculating the proposal ratio. Analyses employing the fossilized birth–death (FBD) model [7] integrate fossil occurrences as data in coestimating time and topology, constraining node ages and, as such, they do not exhibit node age inflation seen in TED analyses that do not employ FBD. While we employ a total evidence approach in our example, combining node and tip calibrations is also applicable to matrices consisting solely of fossil taxa and only morphological characters.

## 5. Conclusion

Nodes and tips are complementary, not competing, approaches to the calibration of molecular clock analyses. Ancient age estimates have become synonymous with tip-calibrated analyses. The construction of the time prior itself is likely to be a causal factor. Our approach to approximating the effective time prior in tip-calibrated analyses shows that when they are implemented alone, tip calibrations can yield divergence time estimates that violate empirical fossil evidence or place exaggerated probability on overly ancient age estimates. Combining node and tip calibrations obviates these effects with the hard minima of node calibrations constraining the uncertainty associated with tip calibrations that, in turn, serve to objectively define the maxima of node age constraints. This approach is appealing because of the positive complementary interaction between the two classes of calibration, but also because it makes the best use of palaeontological data in the construction of evolutionary timescales.

## Supplementary Material

Supplementary Methods

## Data accessibility

The dataset supporting this article is available at: http://dx.doi.org/10.5061/dryad.2q3k2.
