# Supplementary material for: Tips and nodes are complementary not competing approaches to the calibration of molecular clocks
Source: Biol Lett. 2016 Apr;12(4):20150975. doi: 10.1098/rsbl.2015.0975 (PMC4881336; doi:10.1098/rsbl.2015.0975)
Supplement: Supplementary Methods [file rsbl20150975supp1.pdf]

Supplementary Methods - O'Reilly and Donoghue (2016): Tips and nodes are complimentary not competing approaches to the calibration of molecular clocks. *Biology Letters*

Conditional Prior Justification

For a matrix consisting of *s* taxa we can obtain the total number of potential bifurcating, rooted, topologies using

$$\tau_{\text{total}} = \frac{(2s-3)!}{2^{(s-2)}(s-2)!} \ (1),$$

and for any purely bifurcating tree there is a vector *t* of times on the *s*-1 nodes

$$t = [t_1, \ldots, t_{s\text{-}1}]$$

To obtain the effective joint time prior on all possible topologies we must marginalise over topology. For rooted, strictly bifurcating trees this is

$$\mathrm{P}(t) = \sum_{\mathrm{i}=1}^{\tau_{\text{total}}} \mathrm{P}(t|\,\tau_{\mathrm{i}})$$

For a tree of 113 taxa, as we have here, this would involve the unfeasible assessment of the time prior for $\frac{223!}{2^{111}\times 111!}$ bifurcating trees, with an even greater number if allowing for multifurcations. We consider the time prior conditioned on the majority rule consensus tree topology $\mathrm{P}(t|\tau_{\text{cons}})$ a suitable approximation of the time prior as this topology is composed of the most commonly sampled clades, and therefore reflects the distribution of the most commonly sampled time prior.

Divergence Time Estimation - Settings Shared Across All Analyses

Three approaches to divergence time estimation were taken: Node calibration; tip calibration; combined tip and node calibration. For each of these approaches to calibration we obtained a sample of trees from the posterior and a sample of trees from the prior distribution, or in the case of tip-calibration, the "conditional" prior distribution. For all analyses, whether tip, node or combined tip and node calibrated, we utilised the same clock model (IGR), partitioning scheme, partition specific substitution models and prior distributions on parameters associated with these as were used in Ronquist et al. 2012 (3). The uniform tree prior was also employed for all analyses, including node-calibrated analyses, as in the original article. The only priors changed from (3) were the node and tip calibrations, which were swapped for those in (5), with offset exponential distributions assigned to the node calibrations instead of uniform. Offset exponential distributions possess no hard maximum, allowing interaction with other priors to induce maxima on the node calibrations. For all analyses we performed 4 separate runs in MrBayes to assess convergence. These 4 separate runs were combined into a single set of sampled trees; convergence was considered to have been achieved with ESS scores >200, split frequency values of <0.05, and through qualitative assessment of the marginal distributions of parameters.

Node Calibration

A posterior sample of trees was obtained under node calibration by running MrBayes for 20,000,000 generations, sampling every 2000th. A sample of trees from the prior

distribution was obtained by sampling for the same number of generations at the same frequency, but with the `mcmc usedata = NO` setting in MrBayes.

Tip Calibration
Tip calibrated analyses ran for 50,000,000 generations, sampling every 5000th when sampling from the posterior, and 20,000,000 sampling every 200th when sampling from the prior conditioned on the consensus topology.

Tip + Node Calibration
Taxa, both fossil and extant, were assigned to clades based on their placement in the consensus tree produced from the posterior sample of tip-calibrated trees. Topological constraints were then designed to enforce monophyly of these clades, facilitating the application of node calibrations to each clade that was calibrated in the exclusively node-calibrated analyses. As tip-calibration is a relatively new approach to divergence time estimation many aspects of standard phylogenetic algorithms are yet to properly accommodate tip calibration. One such area is the proposal distribution for clade age when there is an overlap between a tip-calibration and the node calibration for the clade to which it is assigned. MrBayes appears to allow for the proposal of tip ages that are older than the clade to which they are assigned, leading to an error when assessing if the new, impossible, state should be accepted in the chain. To deal with this we have discarded any fossil that could conflict with its subtending node calibration, in the case of Tenthredinoidea and Apocrita we have not calibrated the clade as this would require the disposal of a large number of fossil taxa. By enforcing monophyly of clades, but not any more fine scale topological constraints, we allow for the placement of all fossil taxa assigned to a given clade as stem group members, this allows for violation of the minimum bound for our node calibrations. This phenomenon is prominent in Pamphilioidea where a small number of trees in the sample possess all fossils in this clade as stem group members, effectively removing the minimum age constraint for the crown group. This issue highlights the need for non-exclusive constraints that also allow for calibration in MrBayes.

Xyelidae was not assigned a node calibration due to issues highlighted by the original authors with the placement of the fossil used to define the node calibration as a stem and not crown group member. Similarly, in our tip-calibrated analyses *Eoxyela* was often placed outside of crown Xyelidae and further demonstrating its unsuitability as a node calibration for crown Xyelidae.

Tip + Node calibrated analyses ran for 50,000,000 generations, sampling every 5000th when sampling from the posterior, and 20,000,000 sampling every 2000th when sampling from the prior conditioned on the consensus topology.

Node Calibration Prior Retrieval
To obtain the marginal distributions on the ages of the calibrated nodes we used an R script that calculated the age of each clade in each of the trees sampled from the prior (4). Clades were identified on the basis of their extant members, diagnosed from the topology retrieved from the consensus tree produced from the sample of trees from the node calibrated posterior distribution.

Marginal distributions for the ages of the nine calibrated clades employed by the original authors were retrieved using R (4). For any given node, the marginal distribution of that node age can be approximated from the posterior sample of trees, as the age of each clade is a parameter in the phylogenetic model. Clades were identified on the basis of their extant members, determined from the consensus tree

produced from the sample of trees from the node calibrated posterior distribution, in each sampled tree the node that defines the most recent common ancestor of this set of taxa is identified and its age is stored. The distribution of these ages is the marginal distribution of the age of the clade.

Tip Calibration and Tip+Node Calibration Conditional Prior Retrieval

To obtain the conditional prior for tip-calibrated analyses we fix the topology to that of the consensus tree constructed from the posterior sample of tip-calibrated trees. An R script was written that defines all internal nodes within a given tree (including multifurcations) as topological constraints for MrBayes. The prior is then sampled from conditional on this fixed topology; the sample of trees from the prior are obtained by setting `usedata = NO` in MrBayes.

Marginal distributions for the ages of the nine calibrated clades employed by the original authors were retrieved using R (4). For any given node, the marginal distribution of that node age can be approximated from the posterior sample of trees, as the age of each clade is a parameter in the phylogenetic model. Clades were identified on the basis of their extant members, determined from the topology retrieved from a previous node-calibrated analysis of the data (3) and then in each sampled tree the node that defines the most recent common ancestor of this set of taxa is identified and its age is stored. The distribution of these ages is the marginal distribution of the age of the clade.

References

1. Felsenstein J. Number of evolutionary trees. Systematic Zoology. 1978;27(1):27-33.
2. Ronquist F, Teslenko M, van der Mark P, Ayres DL, Darling A, Höhna S, et al. MrBayes 3.2: efficient Bayesian phylogenetic inference and model choice across a large model space. Syst Biol. 2012;61(3):539-42.
3. Ronquist F, Klopfstein S, Vilhelmsen L, Schulmeister S, Murray DL, Rasnitsyn AP. A Total-Evidence Approach to Dating with Fossils, Applied to the Early Radiation of the Hymenoptera. Systematic Biology. 2012;61(6):973-99.
4. R Core Team (2014). R: A language and environment for statistical computing. R Foundation for Statistical Computing, Vienna, Austria. URL http://www.R-project.org/.
5. O'Reilly J, dos Reis M, Donoghue PCJ. 2015 Dating tips for divergence time estimation. *Trends Genet.* 31, 637-650.
